# Combining deep learning with token selection for patient phenotyping from electronic health records

**DOI:** 10.1038/s41598-020-58178-1

**Published:** 2020-01-29

**Authors:** Zhen Yang, Matthias Dehmer, Olli Yli-Harja, Frank Emmert-Streib

**Affiliations:** 1 Predictive Society and Data Analytics Lab, Tampere University, Tampere, Korkeakoulunkatu 10, 33720 Tampere, Finland; 2 Steyr School of Management, University of Applied Sciences Upper Austria, 4400 Steyr Campus, Austria; 3 College of Artificial Intelligence, Nankai University, Tianjin, 300350 China; 4 Department of Biomedical Computer Science and Mechatronics, UMIT-The Health and Life Science University, 6060 Hall in Tyrol, Austria; 5 Computational Systems Biology Lab, Tampere University, Korkeakoulunkatu 10, 33720 Tampere, Finland; 6 Institute of Biosciences and Medical Technology, Tampere University, Tampere, Korkeakoulunkatu 10, 33720 Tampere, Finland; 7 Institute for Systems Biology, Seattle, WA 98109 USA

**Keywords:** Computational biology and bioinformatics, Machine learning, Computational science, Statistics

## Abstract

Artificial intelligence provides the opportunity to reveal important information buried in large amounts of complex data. Electronic health records (eHRs) are a source of such big data that provide a multitude of health related clinical information about patients. However, text data from eHRs, e.g., discharge summary notes, are challenging in their analysis because these notes are free-form texts and the writing formats and styles vary considerably between different records. For this reason, in this paper we study deep learning neural networks in combination with natural language processing to analyze text data from clinical discharge summaries. We provide a detail analysis of patient phenotyping, i.e., the automatic prediction of ten patient disorders, by investigating the influence of network architectures, sample sizes and information content of tokens. Importantly, for patients suffering from Chronic Pain, the disorder that is the most difficult one to classify, we find the largest performance gain for a combined word- and sentence-level input convolutional neural network (ws-CNN). As a general result, we find that the combination of data quality and data quantity of the text data is playing a crucial role for using more complex network architectures that improve significantly beyond a word-level input CNN model. From our investigations of learning curves and token selection mechanisms, we conclude that for such a transition one requires larger sample sizes because the amount of information per sample is quite small and only carried by few tokens and token categories. Interestingly, we found that the token frequency in the eHRs follow a Zipf law and we utilized this behavior to investigate the information content of tokens by defining a token selection mechanism. The latter addresses also issues of explainable AI.

## Introduction

In recent years, biomedical data science and health data science gained considerable interest because the data flood in these fields posses unprecedented challenges^1–3^. An example for such big data are provided by Electronic health records (eHRs). Electronic health records provide digital data containing information of patients about medication, laboratory results, medical imaging, and unstructured data, e.g., in the form of free clinical notes^4,5^. For representing the data in a computer-readable form, parts of eHRs contain data that are represented according to a controlled vocabulary. Examples for such vocabularies are given by the Logical Observation Identifier Names and Codes (LOINC)^6^ for clinical observations and the Digital Imaging and Communication in Medicine (DICOM)^7^ for imaging data. However, for unstructured data, e.g., discharge summaries, there are no overall standards. This makes the analysis of such text data very challenging.

Originally, eHRs were introduced to help hospitals to perform administrative tasks^8^. However, with the help of the Health Information Technology for Economic and Clinical Health Act of 2009, the adoption rate of eHRs has skyrocketed in the past 10 years^9^ and more and more studies are focusing on secondary applications of eHRs for clinical research^10–13^. However, eHRs are difficult to analyze due to their heterogeneous components and high-dimensional structure. In general, the analyses of eHR data utilizes techniques from natural language processing (NLP) in combination with machine learning algorithms^14–16^. In this paper, we focus on the analysis of the unstructured data in eHRs provided by clinical notes of discharge summaries.

One particular application area for eHRs is the so called patient phenotyping^17^. Patient phenotypes are defined based on criteria that describe the medical condition and symptoms of a patient. The task of patient phenotyping is to correctly predict whether a patient has a specific medical condition or is under the risk of developing one. Therefore, properly predicting patient phenotypes from clinical notes is essential for performing patient-related tasks which include improving patient care and for carrying out clinical research.

An important example for an eHR analysis system is the clinical Text Analysis and Knowledge Extraction System (cTAKES)^18^. cTAKES is an open-source natural language processing system that focuses on extracting information from unstructured digital medical records. It aims to process clinical narratives from electronic health records by recognizing and annotating medical related terms in the texts. cTAKES consists of a sentence boundary detector, tokenizer, normalizer, part-of-speech tagger, shallow parser, named entity recognition annotator, status annotator and negation annotator. The system processes input by applying the components and outputs a structure that contains information about all the recognized and annotated entities along with some attributes marking their properties. A study classifying the discharge summary of patients for identifying patients with depression has been conducted in^19^. For their analysis, they used NLP techniques combined with traditional machine learning algorithms. They processed the data by, first, applying a NLP system called MTERMS^20^ to extract related terms for depression symptom, and these terms were later used as features for classification algorithms. They compared the performance between a MTERMS decision tree, SVM, NNge, RIPPER, and C4.5 decision tree and found that a MTERMS decision tree has the best performance. Their work showed that traditional machine learning methods can perform well on a classification task based on unstructured data. However, such methods typically rely heavily on hand-craft features that need to be defined by experts. Interestingly, the algorithms they employed were unable to understand terms that were outside the scope of the predefined medical terms.

In recent years, deep learning methods appeared as an extension to classical artificial neural networks (ANN)^21^. In contrast to ANN^22–24^, deep neural networks consist of a large number of hidden layers instead of just one and they found ample application in a variety of fields^25–29^. Many deep learning architectures for neural networks have been proposed, e.g., multilayer perceptrons (MLPs), recurrent neural networks (RNNs), autoencoders, deep belief networks (DBNs) or convolutional neural networks (CNNs)^30–32^. Especially, CNNs have been dominating the field of computer vision, and many variants have been developed over the years^21^. LeNet-5^33^ is known to be the first model that introduced convolution and pooling layers into a neural network and their paper established the basic components of CNNs. However, it was not until 2012 when the ImageNet 2012 competition has been dominated by a CNN called AlexNet^34^ which won the first place in the image classification. Further remarkable achievements of CNNs are VGGNet^35^, GoogleNet^36^ and ResNet^37^. These approaches study modifications of convolutional kernels and the structure of the networks aiming to make CNNs smaller and more flexible, while improving their performance. In recent years, CNN have also found applications to general natural language processing problems^38–40^ and it has been shown that CNN are good at extracting local position-invariant features from the input for classification tasks^41^. While traditional machine learning methods require usually the manual definition of features, deep learning methods can help experts to save their efforts on defining such hand-craft features.

For analyzing eHRs, deep learning networks have been studied in^42^. Their objective was to predict whether patients qualify for recruitment in a depression study. They built two multilayer feed-forward deep neural network architectures and combined these two networks by passing the results from the first network to the second network. Their work showed that neural networks even with a simple feed-forward architecture are able to perform well in classification tasks on unstructured data. For patient phenotyping, deep learning networks have been studied in^43^. They used a CNN architecture similar to^38^ and trained their network on patient discharge summaries extracted from the MIMIC-III^44^ database. They compared the performance of CNN with some baseline models, and showed that the CNN model outperformed the baseline models. Moreover, they interpreted their model by extracting the most predictive phrases from the CNN. It turned out that their CNN is able to detect some difficult task-related phrases that are even hard to interpret by non-experts. Their work showed that a suitable deep learning algorithm is able to outperform traditional machine learning algorithms by large margins.

Our paper extends the above studies in the following way. First, we introduce a new CNN that processes input from the word and sentence-level. We call this model ws-CNN. That means instead of just using information from the word-level, as studied, e.g., in^38,43,45^, we utilize also semantic information formed by sentences. Second, we study learning curves for the performance of patient phenotyping for ten disorders. This will provide information about the sufficiency of available sample sizes of the training data. Third, we study the influence of (non-random) token selection mechanisms on the classification performance. From this analysis, we gain insights about the semantic contribution of different tokens and token categories. Fourth, we study general characteristics of eHR data with respect to the distribution of token frequencies in discharge summary notes. We will show that the results of this analysis can be utilized for token selection mechanisms. Overall, from the combined results of all our investigation, we will draw general conclusions about deep learning classifiers for patient phenotyping.

This paper is organized as follows. In the next sections, we present all of our numerical results and provide a discussion these. Then we present concluding remarks. At the end of the paper, all methods and data are described we used for our analysis.

## Results

In the following, we present our results. We start by investigating characteristics of the data and the disorders. Then we compare results for different CNN architectures and token selection mechanisms.

### Characteristics of the data

**Figure 1:**
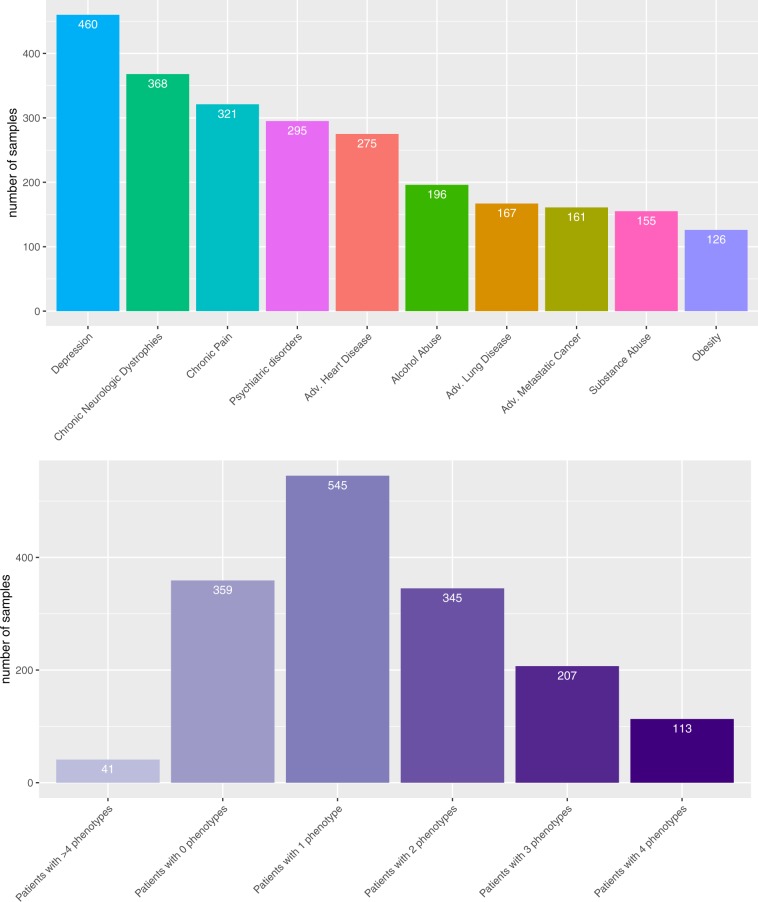
Overview of available patient samples. (**A**) Available samples for the ten disorders. (**B**) Available samples that are categorized to multiple phenotypes.

The text data we use for our analysis contain in total 1, 610 samples, whereas each sample corresponds to a specific discharge summary of a hospital admission for a patient. In Fig. 1A,B, we show an overview of the data. Specifically, Fig. 1A shows the ten disorders we study and the available samples, whereas Fig. 1B shows the number of samples that are labeled by multiple phenotypes. The reason for this is that a patient can suffer from more than one disorder.

After preprocessing the data on the word-level and the sentence-level (see Methods section), we find for a word-level input that the maximum length of a sample consists of 31, 214 characters with 5, 572 tokens. The average number of characters in a sample is 11, 525, and the average number of words in a sample is 2, 067. The smallest sample with the least number of characters consists of 361 characters.

For a sentence-level input, we find the average number of sentences in a sample is 210 and the sample with the least number of sentences consists of 8 sentences. In contrast, the largest sample contains 545 sentences. For all sentences, the longest one contained 150 words, and the average length of a sentence is 9 words. In total, we found 48, 848 different (unique) tokens.

**Figure 2:**
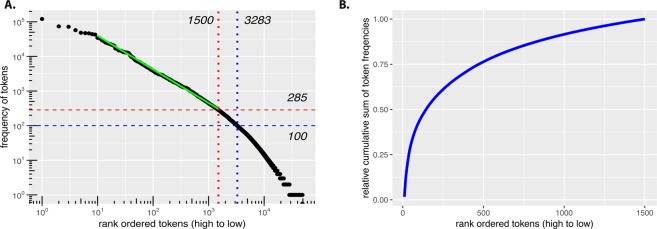
(**A**) Frequency distribution of rank ordered tokens. The solid green line is from a linear regression for the corresponding range showing that the token frequency follows a power law with an exponent of *α* = 0.97. (**B**) Relative cumulative sum of token frequencies for the range of the linear regression fit (10 to 1500) in the left figure.

In Fig. 2A, we show the frequency distribution of the tokens. Specifically, each token appears a certain number of times across all samples and in Fig. 2A we rank ordered the tokens from high to low frequencies. From this figure, we see that only 3284 tokens appear more than 100 times (blue dashed lines), i.e., the vast majority of tokens (45564) has a lower frequency.

In Table 1, we summarize the frequencies of tokens into categories to simplify the interpretation of Fig. 2A. From this table one can see that there are only 20 tokens with a frequency higher than 20, 000. Furthermore, one can see that most of the tokens have frequencies between 1 to 100. Interestingly, there are 20, 293 tokens that only appear once in the whole data set. A further investigation of these tokens reveals that most of these correspond to a sequence of numbers, e.g., ‘1203’, or ‘11850’ while the rest of them are rare vocabularies, spelling errors, or different styles of abbreviations.

**Table 1:** Numbers of tokens per category corresponding to different frequency ranges obtained from Fig. 2.

**Frequencies**	**1**	**2–5**	**5–10**	**10–25**	**25–50**	**50–100**	**100–200**	**200–300**	**300–20,000**	**>20,000**
**Numbers of tokens**	20,293	12,807	4,103	4,065	2,362	1,947	1,280	554	1418	20

For the frequency distribution of rank ordered tokens in Fig. 2A, we perform a linear regression fit for the tokens from rank 10 to 1500. As a result, we find that these frequency values follow a power law  ~ *x*^−*α*^ (solid green line in Fig. 2A) with an exponent of *α* = 0.97. Interestingly, it is know that word frequency distributions of various languages follow a power law^46,47^, which is also known as Zipf’s law^48^. Hence also the tokens of medical notes from hospitals show this behavior.

Furthermore, for the tokens from rank 10 to 1500 we calculate the relative cumulative sum of token frequencies, shown in Fig. 2B. Here we divided the cumulative sum by the total sum of frequencies to obtain a range between zero and one. From this figure one can see that there is a rather gradual accumulation of token frequencies requiring almost all of these tokens to saturate. This indicates the absence of a scale in the data, which is typical for a power law behavior.

From a statistical perspective, it does not make sense to use tokens for a classification that appear only once in the whole data set because they can either be utilized in a training sample or a test sample but not in both. Hence, they cannot help the model to learn or to generalize. A similar argument holds for tokens with a low appearance frequency. Hence, it is natural to remove such tokens from the data. We will return to this important point below in section ‘Token selection: frequency filtering and category prioritization’, when studying the systematic removal of such tokens.

### Balancing classes for training

From Fig. 1A, one can see that the sample sizes per disorder are quite uneven. In order to counteract this potential bias for the training of the neural networks, we used a weight-balancing. That means we pass class weights to our loss function to make learning cost-sensitive. Specifically, we apply a weight-vector to the cross-entropy loss function whereas its components are given by 1/*#*samples per class. This allows the network to penalize false predictions on the minority class in a stronger way. By applying this technique, we observed significant improvements in the performance scores. In Table 2 we show results for the three phenotypes that were effected most by this balancing. For the analyses in the following sections, we always used weight balancing for the training.

**Table 2:** Results of weight-balancing on the performance of three phenotypes. ‘Without’ indicates results without weight-balancing and ‘with’ indicates results with weight-balancing.

Phenotypes	Precision%		Recall%		ROCAUC%		F1%	
	Without	With	Without	With	Without	With	Without	With
Adv. Lung Disease	**80.55**	61.18	35.29	**58.04**	67.07	**76.89**	47.74	**58.90**
	±4.97	±2.93	±3.54	±4.94	±1.71	±2.43	±3.62	±3.68
Chronic Neuro	75.66	71.72	55.80	**66.67**	75.00	**79.31**	62.79	**68.41**
	±2.61	±2.34	±4.63	±3.82	±2.02	±1.78	±3.09	±2.30
Chronic Pain	**77.36**	68.29	35.82	**48.91**	65.93	**71.27**	45.92	**55.96**
	±5.34	±3.76	±4.27	±2.59	±1.53	±1.06	±2.75	±1.69

### Comparison of network architectures

For the following analysis, we perform a binary classification for each of the ten phenotypes. That means in total we train ten different models for the ten phenotypes and each binary classification compares one phenotype against all remaining phenotypes. In this section, we study two different architectures of convolutional neural networks. The first is called w-CNN and the second ws-CNN. As performance metrics for the classification, we report results for the AUROC and F-score^49^.

#### CNN for word-level input

**Figure 3:**
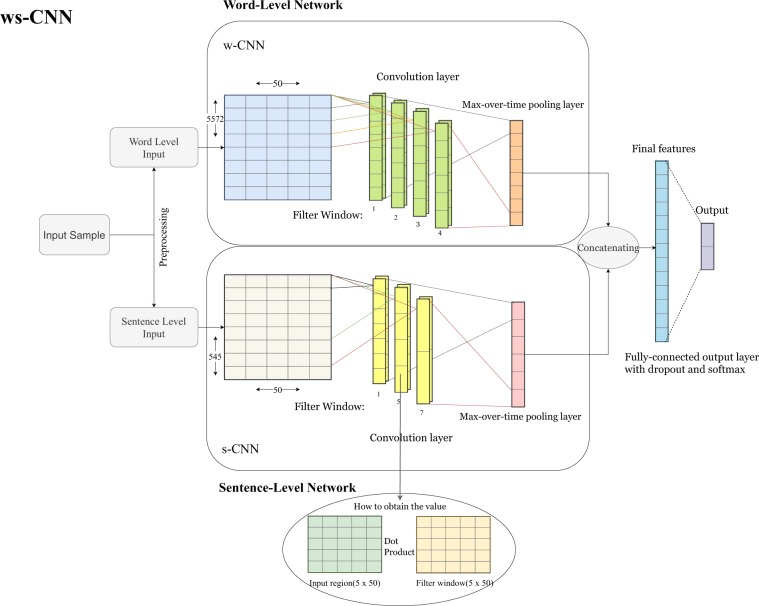
An overview of the architecture of the combined word- and sentence-level convolutional neural network (ws-CNN) which combines a word-level network with a sentence-level network. The architecture of the word-input part (upper network) corresponds also to the architecture of the w-CNN.

The network architecture of the w-CNN is shown in Fig. 3 (upper network). This network uses only information from the word-level as input. The idea is to use a word embedding for tokens but ignore a structuring of discharge summaries as provided by periods or punctuation marks.

**Figure 4:**
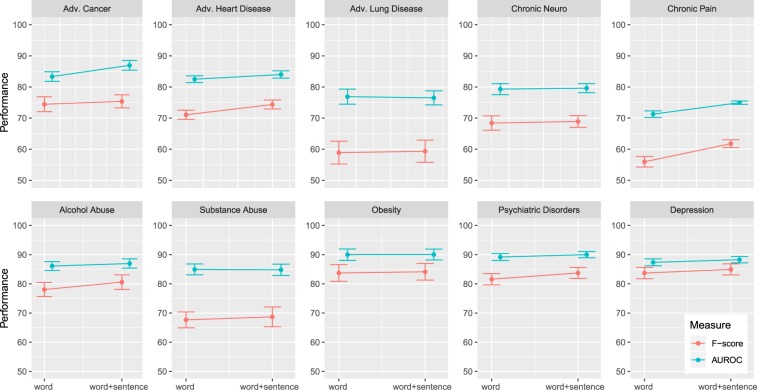
Comparison of the results for the w-CNN and the ws-CNN. For both networks the performance and its standard error is shown. The error bars correspond to the standard error.

 Figure 4 shows the results for the word-level network. The results were obtained using a 10-fold cross-validation and the error bars correspond to the standard error (SE). From the figure one can see that the AUROC is for seven phenotypes 80% or higher and for obesity even 90%. The worst result was obtained for Chronic Pain with slightly over 70%. Similarly, for the F-score six phenotypes obtain 70% or higher. Also here the worst result is obtained for Chronic Pain with slightly over 55% (and an accuracy of 84.72%, not shown). The standard errors were in general around 1.50%, but the largest values were obtained for Adv. Lung Disease (SE of 2.43% for AUROC, 3.68% for F-score).

#### CNN for combined word and sentence-level input

The network architecture of the ws-CNN is shown in Fig. 3 (entire network). This network uses word and sentence-level input. We used summing and averaging pooling for forming the sentence embeddings. Considering that different pooling methods used for the training will result in different performance results, for our analysis we only used the pooling methods that gave the best performances. Specifically, we used summing pooling for training of Adv. Cancer, Adv. Heart Disease, Chronic Pain and averaging pooling for training of Adv. Lung Disease, Chronic Neuro, Alcohol Abuse, Substance Abuse, Obesity, Psychiatric Disorders, Depression.

According to^50^, word embedding representations allow meaningful algebraic operations between word embeddings. However, each sample can contain thousands of tokens, and the inability to search for a bigger neighborhood of words will limit the ability of the network to understand a document. Therefore, we introduced a sentence embedding, which is realized by pooling all the tokens in a sentence. This results in a single vector, we call sentence embedding. The procedure of producing a sentence embedding is essentially a mapping of the whole sentence to the embedding space by algebraic operations between all the words in a sentence.

The results for the combined ws-CNN are shown in the Fig. 4. One can see that there is an overall improvement for all phenotypes in the AUROC and F-score compared to the w-CNN. Specifically, all ten F-scores improve and seven AUROC values (the results for Adv. Lung Cancer, Substance Abuse and Obesity are unchanged). The percentage gain for the F-scores ranges from 0.41% to 5.82% whereas the largest improvement is observed for Adv. Heart Disease and Chronic Pain. For the AUROC, we observe similar improvements with the largest ones for Adv. Cancer and Chronic Pain. The standard errors are in the same order of magnitude as for the w-CNN. Interestingly, also here the largest errors are observed for Adv. Lung Disease (SE of 2.25% for AUROC, 3.57% for F-score). Overall, the performance results for the ws-CNN are never deleterious and mostly beneficial compared to the w-CNN.

A drawback of the more complex ws-CNN model compared to the w-CNN model is its computation time. In average, it takes about 10% longer for obtaining the results performing similar tasks. Furthermore, the storage requirements for the ws-CNN are larger because multiple embeddings for sentences need to be stored. For this reason and the moderate performance gain of the ws-CNN, we focus in the following on the w-CNN to study its characteristics in detail.

### Learning curves

**Figure 5:**
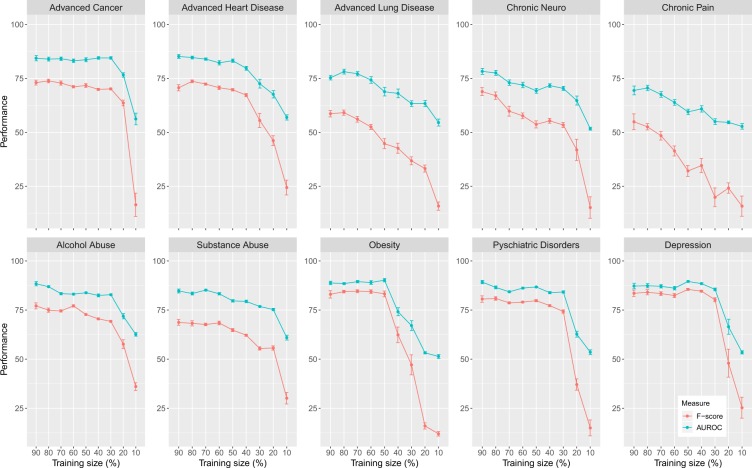
Learning curves of the w-CNN showing the influence of the training sample size. The performance (F-score and AUROC) is shown in dependence on the size of the training data. The error bars correspond to the standard error.

Next, we study learning curves showing the influence of the size of the training data on the classification. We do this by systematically removing a certain percentage of samples from the training data. This is done via a random selection of samples. In Fig. 5, we show the results of this analysis. Here the x-axis shows the percentage of the training samples used for the training. For instance, when using *x* percentage for the training, we remove 1 − *x* percentage of the training samples. That means from left to right the number of samples in the training data decreases. Hence, this allows us to study the breakdown of the learning capabilities of the w-CNN.

From Fig. 5, one observes two different types of behavior. The first type corresponds to a continuous decaying of the performance, whereas the second type is a stepwise decaying. Examples for the first type can be seen for Adv. Lung Disease, Chronic Neuro and Chronic Pain and examples for the second type are Adv. Cancer, Obesity and Depression.

This reveals that for disorders of the first type, the total number of training samples is too small because even a small reduction leads to a deteriorating effect in the performance whereas disorders of the second type the size of the training data is sufficient and even a reduction can be tolerated. However, even for the phenotypes having a continuous decaying of their performance this process is gentle rather than steep.

### Token selection: frequency filtering and category prioritization

Motivated by the above results for the influence of the size of the training data, we study now the influence of non-random data reductions. In the following, we reduce the data by decreasing the number of tokens rather than by reducing the number of samples of the training data. For this we study two different types of non-random token selections. The first type decreases tokens according to their occurrence frequency and the second type performs a prioritization based on token categories.

#### Frequency filtering of tokens

From Fig. 2A, we see that there is a large number of tokens occurring infrequently. For instance, the number of tokens that appear only once in the data is 20, 293, which corresponds to over 42% of all tokens. Most of these tokens were spelling errors or different kinds of abbreviations. In order to study the influence of the tokens on the classification performance, we systematically removed less frequent tokens from the data. That means we remove tokens occurring less or equal frequent than *θ* and we repeated the analysis for the remaining tokens. In Table 3 we show the token threshold *θ* and the corresponding number of tokens removed.

**Table 3:** Token threshold (*θ*) and the number of removed and remaining tokens as a result from this filtering.

**Token threshold***θ*	**1**	**5**	**10**	**25**	**50**	**100**	**200**
**Removed tokens**	20,293	33,102	37,205	41,270	43,632	45,579	46,859
**Remaining tokens**	28,555	15,746	11,643	7,578	5,216	3,269	1,989

**Figure 6:**
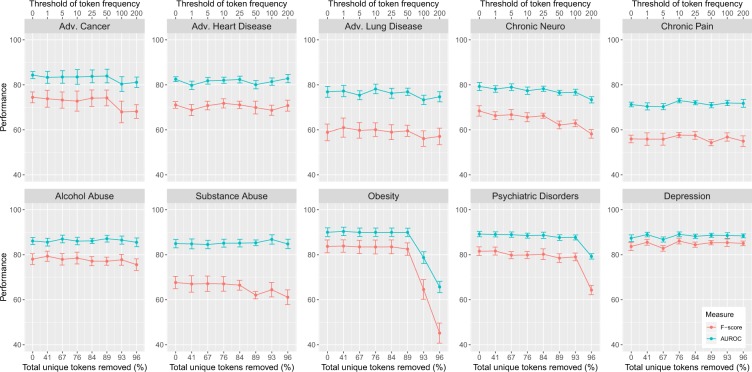
Frequency filtering of tokens. F-score and AUROC for the ten disorders in dependence on high-frequency tokens. Tokens were removed according to their occurrence frequency (from low to high). The upper x-axis give the frequency threshold *θ* while the lower x-axis shows the percentage of remaining tokens.

In Fig. 6, we show the results for the ten disorders for the w-CNN. Overall, one can observe that the performance measures are quite stable up to high thresholding values of *θ*. That means the learning models perform stable with a very limited number of tokens (see lower x-axis). It is clear that at a certain point, the performance breaks down because the information captured by the remaining tokens is no longer sufficient to achieve a good classification performance. Examples for this can be seen for Obesity and Psychiatric Disorders. We stoped our filtering at *θ* = 200 because beyond this point also the performance of the remaining disorders deteriorates. Hence, from our results one can conclude that token filtering up to *θ* = 200 does still lead to good performance results, except for the two aforementioned disorders.

#### Token selection based on category prioritization

In order to perform this analysis, we categorize the tokens into seven categories: symptoms (S), descriptions (D), medicine (M), body related (B), numbers (N), verbs (V) and others (O). These categories have been chosen to represent the semantic meaning of the tokens. That means in contrast to the results in Fig. 6, we now remove tokens with respect to their semantic importance rather than their occurrence frequencies.

**Figure 7:**
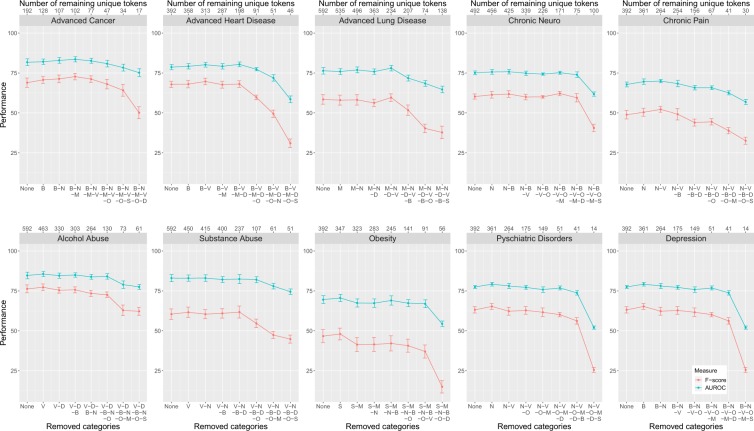
Token selection based on category prioritization. F-score and AUROC for ten disorders in dependence on token categories. Tokens were removed according to categories by using a greedy-strategy. The lower x-axis shows the token categories while the upper x-axis shows the number of remaining tokens.

The results are shown in Fig. 7. Here the x-axis indicates the categories that have been removed (lower axis) and the number of remaining (unique) tokens (upper axis). Specifically, we start by using the tokens from all categories, i.e., none of the categories is removed. Then we remove one category at a time in a greedy-manner by removing the category that has the highest performance. Equivalently, this corresponds to the category that leads to the smallest loss in performance - this gives the same category.

The results in Fig. 7 are interesting because of three reasons. First, using all categories, i.e., removing none of the token categories, shows that a few hundred tokens are sufficient to give very good classification results. For instance, for Adv. Cancer the total number of tokens in all categories is 192. That means by using only 0.39% (192/48, 848) of all tokens the classifier performs almost as good as when using all tokens. Similar results hold for the other disorders. One can observe that the largest number of total tokens in all categories is 592 (for Adv. Lung Disease, Alcohol Abuse and Substance Abuse) corresponding to 1.21% (592/48, 848) of all tokens, which is still a very small proportion of all tokens. Second, all disorders tolerate a further reduction of token categories. For instance, Adv. Cancer, Adv. Heart Disease, Adv. Lung Disease, Chronic Neuro, Alcohol Abuse, Substance Abuse, Psychiatric Disorders and Depression (in total eight disorders) have still an AUROC of over 75% for 17, 91, 234, 171, 61, 51, 41 and 41 tokens respectively. Third, the importance of token categories is heterogeneously distributed. This means that a few categories are more important than others. This can be seen from Table 4 where we summarized the ranks of the categories for the greedy selection in Fig. 7. From this one can see that the category ‘symptoms’ is by far the most important one followed by ‘descriptions’ and ‘others’. It is interesting to note that Obesity behaves differently to all other disorders because ‘symptoms’ seem to be the least informative category. This may be understandable when considering that Obesity is a medical condition that is characterized by an excess accumulation of body fat^51^ which is quite different to the symptoms and etiologies of all other disorders.

**Table 4:** Shown are the ranks for the greedy selection of token categories. The sum represents the total summed up ranks across all phenotypes.

Phenotype/Category	Symptoms	Descriptions	Medicine	Body	Numbers	Verbs	Others
Adv. Cancer	2	1	5	7	6	4	3
Adv. Heart Disease	1	4	5	7	2	6	3
Adv. Lung Disease	1	5	7	3	6	4	2
Chronic Neuro	1	2	3	6	7	5	4
Chronic Pain	1	4	2	5	7	6	3
Alcohol Abuse	1	6	2	5	4	7	3
Substance Abuse	1	4	2	5	6	7	3
Obesity	7	1	6	4	5	2	3
Psychiatric Disorders	1	3	4	2	7	6	5
Depression	1	2	3	7	6	5	4
**Sum**	17	32	39	51	56	52	33

## Discussion

In our study, we addressed the question if a CNN that receives an input beyond the word-level leads to improved classification capabilities. For this reason, we introduced a new CNN that combines information from the word-level and the sentence-level simultaneously, called ws-CNN. As a result, we found that such a ws-CNN improves indeed the classification performance, as shown in Fig. 4, however, the increase in performance is for most of the ten studied phenotypes only moderate. The largest margin of improvement was by 5.82% for the F-score and 5.19% for the AUROC for Chronic Pain. Importantly, this is also the phenotype that is most difficult to classify for the w-CNN. A possible reason for this is that the symptoms of Chronic Pain are rather unspecific that can occur also in other disorders.

For our analysis, we used case-sensitive word embeddings, i.e., we distinguished between upper case and lower case words. In Table 5, we show a comparison between results obtained for case-sensitive (CS) word embeddings and case-insensitive (CI) word embeddings. Overall, the results for all disorders are similar within the standard errors of the error measures, i.e., there is no clear advantage of either word embedding over the other. This is understandable because the word embedding entails similar values for upper and lower case words.

**Table 5:** Results for ws-CNN using case-insensitive (denoted CI) and case-sensitive (denoted as CS) word embeddings. The standard errors of the error measures are shown below the averaged values. The results are obtained from 10-fold CV.

Phenotype	Precision %		Recall %		ROCAUC %		F1 %	
	CI	CS	CI	CS	CI	CS	CI	CS
Adv. Cancer	**81.29**	75.53	71.32	**76.69**	86.62	**86.96**	75.14	**75.37**
	±2.96	±3.62	±3.29	±3.08	±1.54	±1.56	±1.78	±2.10
Adv. Heart Disease	72.68	**77.67**	**74.24**	72.83	**84.28**	84.00	73.04	**74.36**
	±2.59	±3.23	±3.23	±2.86	±1.43	±1.17	±1.81	±1.44
Adv. Lung Disease	**63.93**	63.41	**71.21**	56.83	**83.25**	76.51	**66.99**	59.34
	±1.65	±3.46	±3.30	±4.51	±1.57	±2.25	±1.91	±3.57
Chronic Neuro	70.91	**71.72**	66.62	**67.46**	79.21	**79.62**	68.41	**68.91**
	±2.20	±2.22	±2.99	±3.25	±1.56	±1.45	±2.23	±1.88
Chronic Pain	**67.19**	67.11	**60.13**	58.22	**76.30**	74.96	**63.02**	61.78
	±3.43	±4.21	±2.43	±1.97	±0.80	±0.53	±1.07	±1.25
Alcohol Abuse	81.84	**87.40**	79.55	**88.46**	86.11	**86.95**	80.06	**80.59**
	±3.27	±3.40	±3.69	±3.15	±1.81	±1.59	±2.54	±2.49
Substance Abuse	62.12	**64.71**	**76.75**	74.16	**84.94**	84.81	68.00	**68.68**
	±4.62	±4.05	±3.73	±3.61	±2.05	±1.92	±3.61	±3.39
Obesity	86.06	**88.63**	**84.16**	81.02	**91.44**	90.04	**84.31**	84.13
	±3.86	±3.78	±3.62	±3.69	±1.77	±1.87	±2.40	±2.85
Psychiatric Disorders	79.38	**83.96**	85.02	**89.96**	89.19	**90.00**	81.86	**83.72**
	±2.32	±2.37	±2.47	±1.79	±1.28	±1.08	±1.93	±1.90
Depression	91.18	**92.04**	**82.73**	79.56	**89.34**	88.26	**86.11**	84.94
	±2.18	±1.97	±1.93	±2.38	±0.77	±0.90	±1.04	±1.09

In order to understand the behavior of the sentence-level input processing steps in detail, we conducted additional analyses. An important step of the sentence-level input processing is the definition of a sentence. For this we defined some general rules that set the boundaries for separating sentences from the discharge summary documents. This is a sophisticated process because in free-form texts the writing formats and styles can vary considerably between different records. Therefore, the quality of each separated sentence can vary. From manually examining sentences in some samples, we estimate that about 40% of the separated sentences were perfectly clear and meaningful, 50% are of good quality, while the remaining 10% could benefit from a better processing.

Another processing step is the sentence embedding, which is realized by pooling all the word embeddings in a sentence. Typically in a sentence, not all words contribute equally to the semantic meaning. Instead, words like ‘a’ or ‘the’ can be considered as meaningless tokens for forming the sentence embeddings. We consider a pooling solution an efficient way to preserve the overall meaning of a sentence, although, by the summation or averaging over all the word embeddings in the sentence a certain information loss is inevitable, especially for long sentences. The factors discussed above could be a reason why adding an extra sentence-level input to the network had only a moderate effect on the patient phenotyping.

However, as more important factor that effects the classification performance we consider the *quality* and the *quantity* of the data itself. For this reason, we studied learning curves of the classifiers^52^. From this, we found two types of behavior (continuous decaying and stepwise decaying) of the classification performance (see Fig. 5) indicating that the available sample sizes for the training data are not ideal but sufficient to obtain saturated results. Specifically, for the phenotypes of Adv. Cancer and Obesity the sample sizes are more adequate than for Adv. Lung Cancer and especially Chronic Pain. The latter ones would clearly benefit from large training data.

In order to study the *quality* of the data, we investigated two non-random token selection mechanisms that both reflect on the importance of tokens. The first was based on frequency filtering (Fig. 6) and the second on category prioritization (Fig. 7). From both analyses, we found that there is only a very small fraction of all tokens (between 0.39% and 1.21%) that make a significant contribution to the classification performance. Of course, by adding more tokens (or token categories) the performance increases further, but in a rather shallow way.

If one represents the quantity of the data by the available sample size *N* and the quality of the data by the fraction of informative tokens *p*_*t*_, then it is plausible to assume that the higher the product of both is, i.e., *ψ* = *N* × *p*_*t*_, the more informative are the data. Hence, the value of *ψ* characterizes the overall information content of the data. This metric can be used to characterize the requirements for complexity classes of learning models, e.g., classifiers. For instance, when using complex learning models *M*_*c*_, one needs data that are more informative to learn the model than for simple models *M*_*s*_, i.e., *ψ*_*c*_ > *ψ*_*s*_. That means, complex models have a certain minimal requirement on the information content of data, that can be expressed by the threshold ${\psi }_{c}^{\prime} =N^{\prime} \times {p}_{t}^{\prime}$. If one has data with $\psi  < {\psi }_{c}^{\prime}$ then only simple models can be used. This seems to be the problem in our case. From $N^{\prime} \times {p}_{t}^{\prime} =N\times {p}_{t}$ one obtains the sample size requirement given by $N=\alpha N^{\prime}$ with $\alpha ={p}_{t}^{\prime}$/*p*_*t*_. Hence, for *α* > 1 this can be seen as an effective increase in the sample size over $N^{\prime}$.

There are two major ways to improve the overall information content of data: (1) by increasing the proportion of informative tokens (*p*_*t*_) or (2) by increasing the sample size (*N*). An increase of the proportion of informative tokens could be achieve by improving the vocabulary and its usage utilized by clinicians in the hospitals to write their discharge summaries. However, this would require an extensive training program for the clinicians with considerable associated costs. This is clearly in the realm of health authorities and outside of an experimental design that could be influenced or changed in a rapid way. In contrast, increasing the sample size (by keeping *p*_*t*_ constant) seems more viable and requires merely repositories for eHRs that collect data in a continuous and coordinated manner.

It is interesting to note that also for the classification of image data, tens of thousands (MNIST^33^) or even hundreds of thousands (EMNIST^53^) samples are needed in order to achieve outstanding results^33,54,55^ rather than 1610 samples, as was the sample size in our study. For a traditional statistical analysis such a number of samples would be considered large, however, considering the complexity step involved by going from a w-CNN model to a ws-CNN model for deep learning these numbers need to be reevaluated.

It is interesting to note that a recent systematic review^56^ found that traditional machine learning methods are far more prevalent in clinical settings than deep learning methods. Given the short history of deep learning this is understandable, however, for reasons of comparison we studied the classification performance of a SVM, one of the most widely used classifiers^57^. In Table 6, we show results for a SVM with id-itf (denoted by I) and a SVM with word embedding (denoted by W) as features.

**Table 6:** Results from SVM classification using id-itf and word embedding as features. Here, I denotes SVM with id-itf while W denotes SVM with word embedding. We calculate the average for each word embedding of individual tokens along the word embedding dimension so that each token will only have one mean value as its word embedding feature.

Phenotypes	Precision%		Recall%		ROCAUC%		F1%	
	I	W	I	W	I	W	I	W
Adv. Cancer	92.10	67.97	36.02	16.87	67.84	58.02	50.83	26.39
	±2.86	±9.97	±3.70	±2.80	±1.85	±1.40	±4.15	±4.02
Adv. Heart Disease	90.08	19.21	20.74	18.87	60.10	51.46	33.48	18.93
	±3.49	±1.72	±1.33	±2.28	±0.67	±1.02	±1.86	±1.98
Adv. Lung Disease	86.66	37.29	10.22	12.05	55.07	55.05	17.98	17.89
	±1.83	±8.22	±1.82	±3.10	±0.90	±1.57	±3.03	±4.31
Chronic Neuro	84.91	25.90	20.40	24.42	59.63	51.82	32.55	24.99
	±3.37	±1.93	±1.66	±2.02	±0.88	±1.04	±2.34	±1.84
Chronic Pain	60.83	25.89	06.84	21.49	52.84	53.14	12.21	23.28
	±7.82	±1.69	±1.19	±2.05	±0.62	±0.89	±2.06	±1.80
Alcohol Abuse	95.23	17.81	35.76	13.86	67.77	52.58	51.82	15.38
	±2.43	±2.45	±2.23	±2.22	±1.16	±1.08	±2.67	±2.25
Substance Abuse	95.83	45.47	28.45	16.20	64.16	57.34	42.79	23.45
	±2.84	±8.12	±3.46	±4.04	±1.73	±2.03	±4.55	±5.33
Obesity	60.00	15.51	05.64	06.34	52.82	51.79	10.21	08.71
	±1.63	±4.67	±1.75	±2.39	±0.87	±1.20	±3.08	±2.97
Psychiatric Disorders	79.80	20.98	20.32	18.00	59.62	51.36	31.92	19.13
	±4.03	±2.19	±2.65	±2.25	±1.38	±1.14	±3.64	±2.07
Depression	77.52	31.63	22.86	30.86	60.15	51.82	35.10	31.06
	±4.21	±2.03	±1.97	±1.85	±1.11	±1.32	±2.72	±1.76

Overall, one can see that both SVM classifiers result in inferior results for all disorders compared to W-CNN and ws-CNN. Hence, for our data, a SVM does not offer an alternative way of analysis that would be competitive.

The rational for studying token selection comes from the desire for an explainable AI^58,59^. Frequently, AI (artificial intelligence) is criticized for leading to good prediction results but lacking an interpretation for these. In our case, this means using all words and sentences in discharge summary notes provides us with a mechanism for categorizing disorders, however, without pointing to individual words as the main discriminators. In order to compensate for this shortcoming we performed token selection (for explainable AI this is a simple mechanism to obtain better interpretations for models^60^). Interestingly, we found that, independent of the disorder, a substantial amount of tokens can be removed without a deteriorating classification performance (see Fig. 6). Furthermore, a token categorization revealed the disorder specific importance of these (see Table 4). These results provide at least some degree of explainability for the obtained classification results and are related to the quality of data, discussed above.

Finally, we would like to remark that the idea of investigating different token selection mechanisms to study data quality was inspired by our analysis of the frequency distribution of tokens (Fig. 2). Specifically, the power law behavior (Zipf law^48^) we found suggested this effect implicitly. For general texts of natural langues this effect is well known^47^, but we are not aware that this have been previously reported for eHR discharge summary notes.

## Conclusion

Recent years have seen a drastic increase in the adoption of electronic health records for machine learning and artificial intelligence based analysis systems. In this context, the problem of patient phenotyping from unstructured clinical text records is of particular practical interest because it opens new avenues for quality control in hospitals, the establishment of uniform care standards and clinical research.

In this paper, we shed light on the question why the classification of eHR data with deep learning neural networks leads to good classification results, however, without being groundbreaking as for image or audio data^21,61,62^. We found that the combination of data quality and data quantity of the text data is playing a crucial role. As a summary from our investigations, we conclude that in order to introduce more complex network architectures that improve significantly beyond the w-CNN model one requires larger sample sizes because the amount of information per sample is very small and only carried by a very small number of tokens. Due to the fact that only health authorities cannot directly influence the way clinicians are recording their patient notes (which would effect the quality of the data) a practical experimental design action is to effect the quantity of the data by increasing the sample sizes of eHR notes.

We think that eHR could be a valuable source of information toward establishing personalized and precision medicine by providing complementary information to genomics and genetics data because only the integration of information across multiple levels will result in a comprehensive systems medicine understanding^63–66^.

## Materials and Methods

In this section, we discuss the data we use for our analysis, the network architectures, and all the necessary preprocessing steps.

### Data

#### MIMIC-III database

For our analysis, we use data from the Medical Information Mart for Intensive Care (MIMIC-III) database^44^. MIMIC-III is a freely accessible database that contains eHRs information from the Intensive Care Unit (ICU) for 53, 423 different hospital admissions for adult patients gathered from 2001 to 2012. The data were collected at the Beth Israel Deaconess Medical Center in Boston, Massachusetts (USA). MIMIC-III is a powerful database since it is the only free and accessible critical care dataset, and it contains data from more than a decade. The information inside MIMIC-III is detailed and specific. The data in the MIMIC-III database ranges from structured data using controlled vocabularies to free-text data such as clinical notes and text interpretations of images studies^44^. In total, MIMIC-III consists of 8 different classes of de-identified data, briefly described in Table 7.

**Table 7:** The MIMIC-III database provides eight data categories.

Data category	Description
Billing	Coded data recorded primarily for billing and administrative purposes. Includes Current Procedural Terminology (CPT) codes, Diagnosis-Related Group (DRG) codes, and International Classification of Diseases (ICD) codes.
Descriptive	Demographic detail, admission and discharge times, and dates of death.
Dictionary	Look-up tables for cross referencing concept identifiers (for example, International Classification of Diseases (ICD) codes) with associated labels.
Laboratory	Blood chemistry, hematology, urine analysis, and microbiology test results.
Medications	Administration records of intravenous medications and medication orders.
Notes	Free text notes such as provider progress notes and hospital discharge summaries.
Physiologic	Nurse-verified vital signs, approximately hourly (e.g., heart rate, blood pressure, respiratory rate).
Reports	Free text reports of electrocardiogram and imaging studies.

#### Discharge summaries from MIMIC-III

According to^67^, among all the data classes, discharge summaries hold the most valuable information for patient phenotyping. For this reason, we focus in this paper on data representing discharge summaries. The MIMIC-III database provides 52, 746 discharge notes for 46, 146 unique patients. Each note has a free-form discharge summary and unique identifiers which include subject ID, admission ID and chart time.

#### Annotated dataset

The goal of this paper is to study the automatic patient phenotyping. For this reason, we need annotated discharge summaries that categorize them into different disorder categories. Due to the fact that the MIMIC-III database does not provide this information we used expert annotated data from^43^. This study provides in total 1, 610 samples annotated in 10 different phenotypes; see Fig. 1A. All these 1, 610 notes were annotated by clinical researchers and medical residents (in total 7) whereas each discharge summary was annotated by at least two annotators. When there was disagreement between annotators, a senior clinicians made the final decision. Importantly, each patient can be annotated with multiple phenotypes. The number of samples for each phenotype range from 126 to 460; for an overview see Fig. 1B.

### Processing pipeline

The samples extracted from the MIMIC-III are stored in a comma-separated values (csv) file, and the discharge summary fields are string format. Discharge summaries are unstructured data, although most of the medical related terms are coded according to ICD-9, ICD-10, or Systematized Nomenclature of Medicine-Clinical Terms (SNOMED CT)^8^. The overall writing can contain spellings errors, typing errors, acronyms, and abbreviations. Furthermore, the writing styles may vary a lot according to personal preferences. Therefore, a natural language processing pipeline is needed before the data can be used as input for the neural networks to be studied. For our analysis, we require two different types of preprocessing, one is for word-level input and another for sentence-level input. In this following subsections, we will describe both preprocessing steps.

### Preprocessing for word-level input

In our experiment, the preprocessing was done using python. The data set we used contained 1,610 total samples, and each sample contained normally a different length of string characters, the data can be expressed as *Data* = {*S*_1_, *S*_2_, *S*_3_, *S*_4_. . . . *S*_1610_}, *S*_*n*_ ∈ [*String*].

Word level preprocessing processes the whole texts of a sample, thus the sentence or paragraph structures are not considered in this case. Sentence level preprocessing will be introduced later in this section.

An overall pipeline of word level preprocessing consists of cleansing, tokenizing, indexing and padding.

#### Cleansing

Cleansing steps aim to remove all the meaningless characters regarding our model. In this thesis, we followed the preprocessing step in previous work^43^. Characters for alphabets and numbers, and some special characters like, () ! ?′ were kept, other characters were removed, since other characters were considered useless in our model other than those characters that we kept.

We used python script to replace all the characters to be removed with a single empty space (including empty space itself), and multiple continuous targets were replaced with a single empty space. This was important because when we were converting each token to its integer representation, the algorithm recognized each individual token by spaces.

#### Tokenization, indexing and padding

Tokenization is the process of setting the boundaries for each individual unit (token). An token can be a word, a sequence of numbers, or a special character. After tokenization, each token was recorded into a lookup dictionary with the index indicating its position in the dictionary. The order of the dictionary can be arbitrary, but in our experiment the dictionary was constructed according to the order of the appearances of the tokens in the samples. The index starts from 1, 1 is the index for token ‘unk’, which is assigned to the token that appeared in the samples but did not exist in the pretrained word embedding. 2 is the index for ‘padding’ token, which is used to pad the samples to a fixed length. There were in total 48,848 different tokens in our experiments, hence the size of the lookup dictionary was 48,848.

Tokenization was followed by indexing procedure which is to replace each token with its index in the lookup dictionary, then each token will be represented as a categorical value. These values are later used in the word embedding lookup step to be replaced by their corresponding word embedding representations.

The last step is padding process. Due to the fact that lengths of the text in different samples may vary, and for the sake of convenience, all the samples with less than the maximum length were padded with integer 2 which indicates the token ‘padding’ in the lookup dictionary. The sample with longest length had 5,572 tokens. Therefore, all the other samples should be padded to have 5,572 tokens. The padding step is different for word and sentence level inputs, this will be discussed in the coming subsections.

Passing the samples through tokenization, indexing, and padding will produce a vector *S* = {*s*_1_, *s*_2_, *s*_3_, *s*_4_. . . . *s*_1610_}, *s*_*n*_ = {*x*_1_, *x*_2_, *x*_3_, *x*_4_. . . . *x*_5572_}, *x*_*n*_ ∈ [1,48848], and this is the structure of the input samples to the word level network.

### Preprocessing for sentence-level input

In our work, we considered sentence level input in addition to word level input which consists of word embeddings, hence we used also input that comprises sentence embeddings (sentence embedding will be introduced later). The working pipeline for preprocessing sentences introduces one more step which is sentence segmentation. Overall, the pipeline for preprocessing sentence-level input consists of: sentence segmentation, cleansing, tokenizing, indexing and padding.

#### Sentence segmentation

Sentence segmentation is a challenging task, because in a free-form text, the sentence breaks do not always follow common rules. Some of the sentences are properly written and end with a period, however, other sentences do not. In order to separate the sentences, we need to define separating rules to specify the boundaries of sentences besides using a period. For our analysis, we also considered commas as a boundary to be separated when the tokens between two boundaries is greater than 5.

After specifying the boundaries, we trimmed the whole text into different sentences for each sample. Hence, a sample is represented as a list of different sentences. For comparing the structure of both level inputs, in a sample that contains word-level input the information is represented as *Sample* = {*Word*_1_, *Word*_2_, *Word*_3_. . . . *Word*_*n*_}, *Word*_*n*_ ∈ [*String*]. In contrast, for a sample that contains sentence-level input, the structure is *Sample* = {*Sentence*_1_, *Sentence*_2_, *Sentence*_3_. . . . *Sentence*_*n*_}, *Sentence*_*n*_ ∈ [*String*] with *Sentence* = {*Word*_1_, *Word*_2_, *Word*_3_. . . . *Word*_*n*_}, *Word*_*n*_ ∈ [*String*].

#### The remaining preprocessing steps for sentence level input

Tokenization and indexing procedures are identical to the ones from word level preprocessing. For the padding step, each sentence may have different lengths of words, also each sample may have different lengths of sentences. Thus padding needs to be done in the sentence dimension as well as word dimension. In our work, the sentence with the maximum length among all the sentences has a length of 150, and the sample with most sentences has a number of 545 sentences. So in our experiment we padded all the sentences to have 150 tokens, and we used padding sentence which is an empty sentence (a sentence consists of 150 ‘padding’ tokens) to pad into each sample until every sample has 545 sentences.

### Word embedding

Traditional natural language processing algorithms treat each word or token as a unitless term, whereas each unit is represented by an unique symbol^68^. Examples of such representations are one-hot vector or n-gram. However, such methods do not take the semantic and syntactic meaning of individual words into account. For this reason, we are using a word embedding. The idea of word embedding was proposed by^69^ and many approaches have been proposed for learning word embeddings from data^70–72^. Such methods combine language modeling and feature learning techniques to project words into a distributed vector space where syntactic and semantic meanings of the words are captured.

The CNNs we use utilize word and sentence embeddings based on word2vec^68^. Word2vec is a well-known approach to train word embeddings that is also relatively fast compared with other approaches. It has two basic models, continuous Bag-of-Words (CBOW) model and continuous Skip-gram model (SG). Both use neural network architecture with in total 3 layers. CBOW model tries to predict the current word based on its neighborhood words using a sliding window, while the SG model predicts the surrounding words from the position of the input word also using a sliding window. The input to the network is a sequence of words, while the middle layer projects the corresponding words within the sliding window to the word embedding dimension. The output layer calculates the probability distribution for each word to appear in the corresponding position. The goal of the network is to optimize the parameters in the network. Those parameters are later used to construct the word embedding dictionary where different words can be mapped into their vector representation using their unique token ID to lookup from the dictionary.

We trained the word embeddings on all the discharge notes from the MIMIC-III database. For the learning we used the CBOW model. The lookup window size was set to 10, and the model filtered the words appearing less than 5 times. Also for every positive example, 10 negative examples were sampled. The model was trained for 15 iterations. The total number of vocabularies in the word embedding lookup table that was trained on the whole discharge notes was 470, 856, while the number of vocabularies for the 1, 610 samples we used for our analysis was 48, 848. The word embeddings we used were case-sensitive. Therefore, the same words with upper or lower cases were assigned with different word embeddings.

### Network architecture

In this paper, we study two different types of networks. The first is a convolutional neural network on the word-level (w-CNN)^15,38^ and the second is a combination of word-level and sentence-level convolutional neural networks (ws-CNN) introduced in this paper.

#### Basic structure

The idea behind the CNN architecture introduced in^15,38^ is to learn different features from combinations of adjacent words using varying sizes of convolutional windows. The convolutional windows operate only along the temporal dimension because there is no meaningful spatial dimension for text data. The basic structure of the CNN we use is shown in Fig. 3.

For such a CNN, the input is a matrix where the vertical dimension is the number of words and the horizontal dimension provides a word embedding. That means each row represents the embedding for a token, i.e, *x*_*i*_ ∈ IR^*d*^, where *d* denotes the dimension of word embedding which was 50 in our study. Convolutional windows will go through the temporal dimension using a stride of 1. Convolutional windows have a dimension of *w*_*i*_ ∈ IR^*i*×*d*^, *i* ∈ {1, 2, 3, . . . *n*}, here *i* denotes the filter length of the convolution window. Each convolutional operation between the filter window the receptive field will produce a single value inside the corresponding feature map. Different feature maps represent different relations extracted from the convolution operations over a neighborhood of words. This is essentially a process of looking for different n-grams from the input.

Various convolutional filters operate on the same input in parallel. Therefore, each group of convolution filters with the same length constitutes a single convolutional layer. Each convolution layer produces *n* feature maps, and each feature map has different lengths of values according to the filter size. Assuming that the length of the filter window is *l*, then each feature value inside a feature map is calculated as *c*_*i*_ = *f*(*w* ⋅ *x*_*i*:*i*+*l*−1_ + *b*). Here *b* denotes the bias term, *x*_*i*:*i*+*l*−1_ denotes the corresponding region processed by the filter and *f* is the activation function. For our analysis we used ReLu as activation function. Then each feature map is given by *C*_*i*_ = {*c*_1_, *c*_2_, *c*_3_. . . *c*_*n*−*l*+1_}, whereas *i* denotes the number of feature maps.

After the convolution layer comes a max-over-time pooling layer. Max-over-time pooling takes only the maximum value from a feature map, which results in a single value for each feature map. It is argued that getting the maximum activation value from the feature maps is most important for the semantics in text mining^15^. During the pooling procedure all of the values pooled from the feature maps are concatenated together to form a vector. For *L* convolution layers each having *n* feature maps, then after pooling the penultimate layer will have a feature vector *V* = {*v*_1_, *v*_2_, *v*_3_. . . *v*_*L*×*n*_} with *v*_*i*_ ∈ IR. Here *v*_*i*_ are the pooled values from max-over-time pooling.

The last layer consists of two neurons providing the final probability distribution of class +1 and class  −1 labels. Specifically, the output is given by *y* = Φ(*w* ⋅ *V* + *b*), where *w* are the parameters between the last and previous layer, *b* is the bias and *V* is the final feature vector. Since we are dealing with a binary classification task, we chose a Sigmoid, Φ, (also called soft-step) as the activation function.

### Combined ws-CNN

The main network we study combines two CNNs^73^. One CNN for the word-level and another CNN for the sentence-level input. The penultimate layer from the combined network concatenates the feature output from the word-level and sentence-level network. Figure 3 shows the architecture of the combined network, we call word-sentence-level convolutional neural network (ws-CNN).

#### Word-level network and word embedding

For the word-level network, we use a similar architecture as in^38,43^. The network receives preprocessed word-level input where each sample, *x*, is a vector of integer values. For our data, the maximum text length of a sample consists of *L* = 5, 572 tokens, hence, each sample is represented by *x* = (*t*_1_, *t*_2_, *t*_3_, …, *t*_*L*_) with *t*_*i*_ ∈ {1, …, 48848} and *i* ∈ {1, …, *L*}. If a sample contains less than *L* tokens, a padding is used fill up these components.

The first layer of the network is an embedding layer where each component (token) of *x* is mapped into a vector of real numbers of dimension 50. Hence, the embedding layer is a two-dimensional matrix *E* of dimensions *E* ∈ IR^5572×50^. All the weights in the embedding layer are trainable. The network will update these values, consequently the embedding lookup table will be updated. We found that by keeping the embedding weights trainable we achieve a better performance than by keeping them fixed. After the embedding layer, the input enters the neural network as shown in Fig. 3.

#### Sentence-level network and sentence embedding

**Figure 8:**
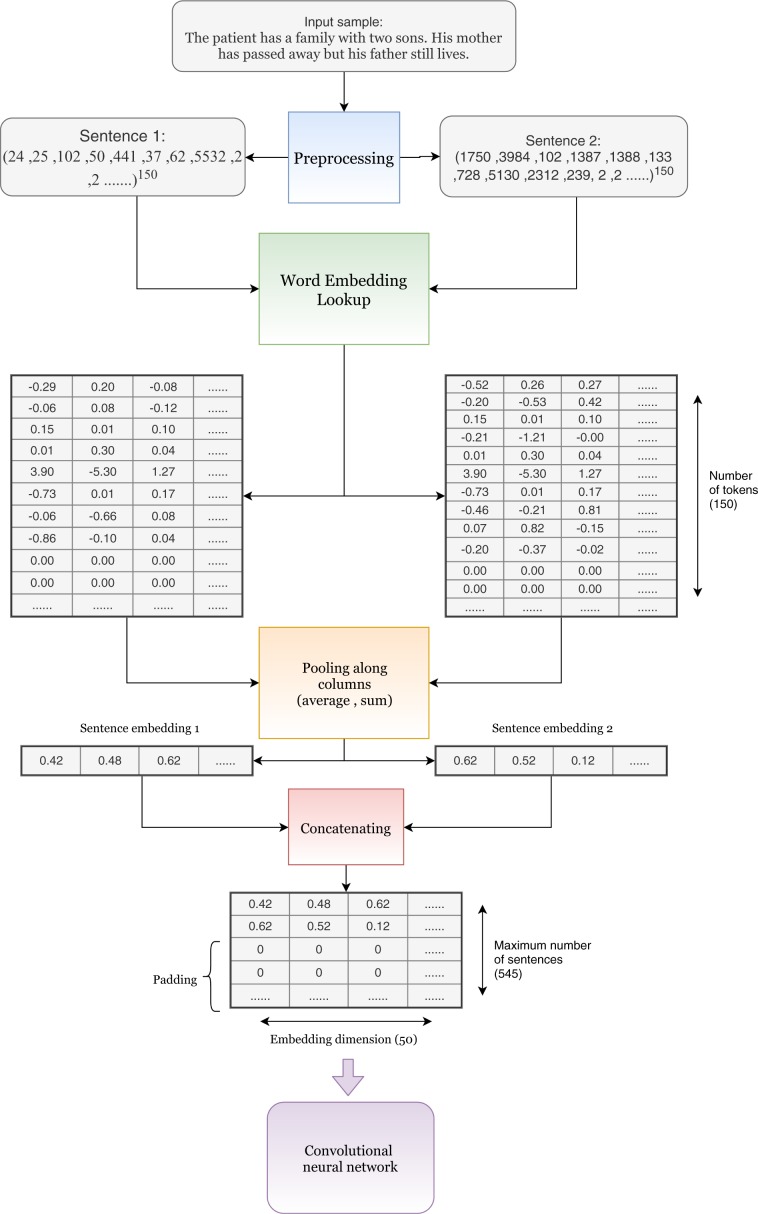
An illustration how we generate sentence-level embeddings. In this example, the input sample consists of two sentences.

For our data, we found that the maximal number of sentences per sample is 545. Furthermore, the maximal number of tokens per sentence is 150. Hence, the sentence-level network receives a preprocessed sentence level input *z*, whereas each sample is represented by *z* = {*s*_1_, *s*_2_, *s*_3_. . . *s*_545_}, *s*_*i*_ ∈ *t*^150^, *t*_*j*_ ∈ [1,48848]. Also an embedding layer is applied to the sentence-level input. In the embedding layer the input *z* is transformed into a 3-dimensional matrix ${E}^{^{\prime} }\in {IR}^{545\times 150\times 50}$. In order to utilize the underlying features in different sentences in an efficient way, we pooled the matrix $E^{\prime}$ by either averaging or summing along the second dimension. It is important to note that the padding tokens in each sentence will not contribute to the pooling operation. Instead, the pooling is calculated for the original length for each sentence before padding. After pooling, the matrix for each sample will be *E*_*p*_ ∈ I*R*^545×50^, here 545 is the number of sentences. Each sentence in a sample is pooled to a vector called sentence embedding, which has a dimension of 50 (same dimension as for the word embedding). Figure 8 shows how to obtain a sentence embedding for a sample.

### Training

The weights for the convolutional layers were initialized using a uniform distribution from the range  −0.01 to 0.01. The filter lengths we used vary with the phenotype. Specifically, we used 1–4 for Adv. Cancer, Chronic Neuro, Depression, 2–5 for Adv. Lung Disease, Chronic Pain, Alcohol Abuse, Obesity, Psychiatric Disorders and 1–5 for Adv. Heart Disease.

Each filter group (this refers to a group of filters with the same length) resulted in 100 feature maps. The parameters of the feature maps were normalized to a norm of 3, and the dropout rate was 0.5. We calculated the cross-entropy to estimate the loss, and we used Adadelta^74^ as our optimizer with a learning rate of 0.5. Specifically, we used a batch-size of 32 and ran each model with 20 epochs. The word embedding weights were further fine-tuned with the gradients passed only from the word-level network during backward pass.

The word-level network in the combined network uses the same input, parameters and filter window lengths as the w-CNN. The sentence-level network uses also the same hyper-parameters except for the filter window lengths and the number of feature maps. For the sentence-level network, we use for all phenotypes filter window lengths of {1, 5, 7, 9} and the number of feature maps was set to 50 for each filter group. The embeddings were shared between both word- and sentence-level network, but only the network for the word-level input will pass gradients to update the word embeddings during training. This helped to reduce the training time of the combined network considerably.

### Overfitting

**Figure 9:**
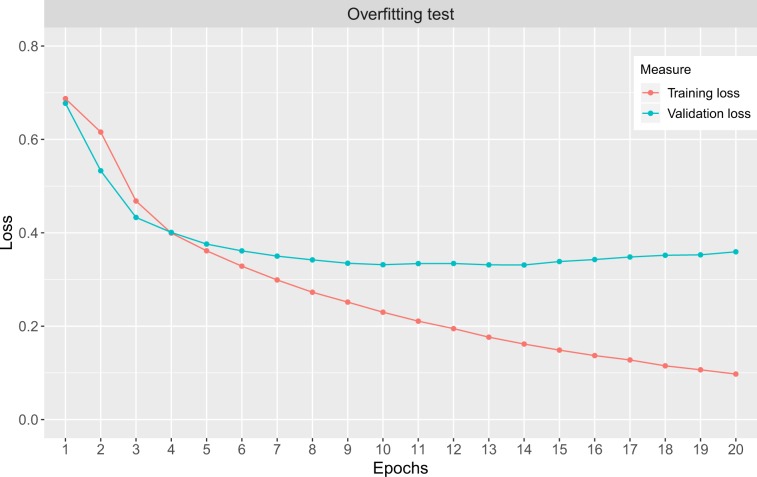
Shown is the loss as a function of epochs. The red curve corresponds to the loss for training data and the red curve for validation data (not used for training). The curves are averaged over all disorders.

In order to identify the possibility of overfitting, we study the loss of training and validation data as a function of epochs for the training of the neural networks. In Fig. 9, we show an example of a ws-CNN for the loss of training data (green) and validation data (red). The curves are averaged over all disorders. As one can see, there is no noticeable effect of overfitting because the validation curve (shown in green) does not increase towards higher epochs. That means stoping the learning either after epoch 8 or 20 does change the training error but not not validation error, which is the approximation of the generalization error^52^. The results for the individual disorders and the w-CNN look similar (not shown).

### Resampling

All results were obtained using a 10-fold cross-validation (CV)^49^. From these results, the average error and the standard error (SE) is obtained.

## References

[1] Cleveland WS (2001). Data science: an action plan for expanding the technical areas of the field of statistics. International statistical review.

[2] Dunn MC, Bourne PE (2017). Building the biomedical data science workforce. PLoS biology.

[3] Emmert-Streib F, Dehmer M (2019). Defining data science by a data-driven quantification of the community. Machine Learning and Knowledge Extraction.

[4] Jha AK (2009). Use of electronic health records in us hospitals. New England Journal of Medicine.

[5] Häyrinen K, Saranto K, Nykänen P (2008). Definition, structure, content, use and impacts of electronic health records: a review of the research literature. International journal of medical informatics.

[6] Huff SM (1998). Development of the logical observation identifier names and codes (loinc) vocabulary. Journal of the American Medical Informatics Association.

[7] Mildenberger P, Eichelberg M, Martin E (2002). Introduction to the dicom standard. European radiology.

[8] Jensen PB, Jensen LJ, Brunak S (2012). Mining electronic health records: towards better research applications and clinical care. Nature Reviews Genetics.

[9] Birkhead GS, Klompas M, Shah NR (2015). Uses of electronic health records for public health surveillance to advance public health. Annual review of public health.

[10] Miotto R, Weng C (2015). Case-based reasoning using electronic health records efficiently identifies eligible patients for clinical trials. Journal of the American Medical Informatics Association.

[11] Tatonetti NP, Patrick PY, Daneshjou R, Altman RB (2012). Data-driven prediction of drug effects and interactions. Science translational medicine.

[12] Doshi-Velez F, Ge Y, Kohane I (2014). Comorbidity clusters in autism spectrum disorders: an electronic health record time-series analysis. Pediatrics.

[13] Knake LA (2016). Quality of ehr data extractions for studies of preterm birth in a tertiary care center: guidelines for obtaining reliable data. BMC pediatrics.

[14] Manning, C. D., Manning, C.D. & Schütze, H. *Foundations of statistical natural language processing* (MIT press, 1999).

[15] Collobert R (2011). Natural language processing (almost) from scratch. Journal of machine learning research.

[16] Ohno-Machado L, Nadkarni P, Johnson K (2013). Natural language processing: algorithms and tools to extract computable information from ehrs and from the biomedical literature. Journal of the American Medical Informatics Association.

[17] Parr DG (2011). Patient phenotyping and early disease detection in chronic obstructive pulmonary disease. Proceedings of the American Thoracic Society.

[18] Savova GK (2010). Mayo clinical text analysis and knowledge extraction system (ctakes): architecture, component evaluation and applications. Journal of the American Medical Informatics Association.

[19] Zhou L (2015). Identifying patients with depression using free-text clinical documents. Studies in health technology and informatics.

[20] Zhou, L. *et al*. Using medical text extraction, reasoning and mapping system (mterms) to process medication information in outpatient clinical notes. In *AMIA Annual Symposium Proceedings*, vol. 2011, 1639 (organizationAmerican Medical Informatics Association, 2011). PMC3243163 22195230

[21] LeCun Y, Bengio Y, Hinton G (2015). Deep learning. Nature.

[22] van Gerven, M. & Bohte, S. *Artificial neural networks as models of neural information processing* (Frontiers Media SA, 2018). 10.3389/fncom.2017.00114 PMC5742181 29311884

[23] Emmert-Streib F (2006). Influence of the neural network topology on the learning dynamics. Neurocomputing.

[24] Emmert-Streib F (2006). A heterosynaptic learning rule for neural networks. International Journal of Modern Physics C.

[25] Alipanahi B, Delong A, Weirauch MT, Frey BJ (2015). Predicting the sequence specificities of dna-and rna-binding proteins by deep learning. Nature biotechnology.

[26] Smolander J, Stupnikov A, Glazko G, Dehmer M, Emmert-Streib F (2019). Comparing biological information contained in mrna and non-coding rnas for classification of lung cancer patients. BMC Cancer.

[27] Litjens G (2017). A survey on deep learning in medical image analysis. Medical image analysis.

[28] Zhang S (2015). A deep learning framework for modeling structural features of rna-binding protein targets. Nucleic acids research.

[29] Schmidhuber J (2015). Deep learning in neural networks: An overview. Neural networks.

[30] Graves, A., Mohamed, A.-R. & Hinton, G. Speech recognition with deep recurrent neural networks. In *Acoustics, speech and signal processing (icassp), 2013 ieee international conference on*, 6645–6649 (organization IEEE, 2013).

[31] Emmert-Streib F (2005). Active learning in recurrent neural networks facilitated by an hebb-like learning rule with memory. Neural Information Processing - Letters and Reviews.

[32] Vu, N. T., Adel, H., Gupta, P. & Schütze, H. Combining recurrent and convolutional neural networks for relation classification. *arXiv preprint arXiv:1605.07333* (2016).

[33] LeCun Y (1998). Gradient-based learning applied to document recognition. Proceedings of the IEEE.

[34] Krizhevsky, A., Sutskever, I. & Hinton, G. E. Imagenet classification with deep convolutional neural networks. In *Advances in neural information processing systems*, 1097–1105 (2012).

[35] Simonyan, K. & Zisserman, A. Very deep convolutional networks for large-scale image recognition. *arXiv preprint arXiv:1409.1556* (2014).

[36] Szegedy, C. *et al*. Going deeper with convolutions. In *Proceedings of the IEEE conference on computer vision and pattern recognition*, 1–9 (2015).

[37] He, K., Zhang, X., Ren, S. & Sun, J. Deep residual learning for image recognition. In *Proceedings of the IEEE conference on computer vision and pattern recognition*, 770–778 (2016).

[38] Kim, Y. Convolutional neural networks for sentence classification. In *Proceedings of the 2014 Conference on Empirical Methods in Natural Language Processing (EMNLP)*, 1746–1751, 10.3115/v1/D14-1181 (Association for Computational Linguistics, Doha, Qatar, 2014).

[39] Che, Z., Cheng, Y., Sun, Z. & Liu, Y. Exploiting convolutional neural network for risk prediction with medical feature embedding. *arXiv preprint arXiv:1701.07474* (2017).

[40] Suo, Q. *et al*. Personalized disease prediction using a cnn-based similarity learning method. In *2017 IEEE International Conference on Bioinformatics and Biomedicine (BIBM)*, 811–816 (organization IEEE, 2017).

[41] Yin, W., Kann, K., Yu, M. & Schütze, H. Comparative study of cnn and rnn for natural language processing. *arXiv preprint arXiv:1702.01923* (2017).

[42] Geraci J (2017). Applying deep neural networks to unstructured text notes in electronic medical records for phenotyping youth depression. Evidence-based mental health.

[43] Gehrmann S (2018). Comparing deep learning and concept extraction based methods for patient phenotyping from clinical narratives. PloS one.

[44] Johnson AE (2016). Mimic-iii, a freely accessible critical care database. Scientific data.

[45] Hu, B., Lu, Z., Li, H. & Chen, Q. Convolutional neural network architectures for matching natural language sentences. In *Advances in neural information processing systems*, 2042–2050 (2014).

[46] Li W (1992). Random texts exhibit zipf’s-law-like word frequency distribution. IEEE Transactions on information theory.

[47] Piantadosi ST (2014). Zipf’s word frequency law in natural language: A critical review and future directions. Psychonomic bulletin & review.

[48] Zipf, G. K. *Human Behaviour and the Principle of Least Effort* (Addison-Wesley, Reading, MA, 1949).

[49] Emmert-Streib, F., Moutari, S. & Dehmer, M. A comprehensive survey of error measures for evaluating binary decision making in data science. *Wiley Interdisciplinary Reviews: Data Mining and Knowledge Discovery* e1303 (2019). 10.1002/widm.1303 PMC6777486 31656552

[50] Mikolov, T., Sutskever, I., Chen, K., Corrado, G. S. & Dean, J. Distributed representations of words and phrases and their compositionality. In *Advances in neural information processing systems*, 3111–3119 (2013).

[51] Pi-Sunyer FX (2000). Obesity: criteria and classification. Proceedings of the Nutrition Society.

[52] Emmert-Streib F, Dehmer M (2019). Evaluation of regression models: Model assessment, model selection and generalization error. Machine Learning and Knowledge Extraction.

[53] Cohen, G., Afshar, S., Tapson, J. & van Schaik, A. Emnist: an extension of mnist to handwritten letters. *arXiv preprint arXiv:1702.05373* (2017).

[54] Jarrett, K. *et al*. What is the best multi-stage architecture for object recognition? In *2009 IEEE 12th international conference on computer vision*, 2146–2153 (IEEE, 2009).

[55] Cireşan, D., Meier, U. & Schmidhuber, J. Multi-column deep neural networks for image classification. *arXiv preprint arXiv:1202.2745* (2012). 10.1016/j.neunet.2012.02.023 22386783

[56] Sheikhalishahi S (2019). Natural language processing of clinical notes on chronic diseases: Systematic review. JMIR medical informatics.

[57] Vapnik, V. N. *The Nature of Statistical Learning Theory* (Springer, 1995).

[58] Holzinger, A., Biemann, C., Pattichis, C. S. & Kell, D. B. What do we need to build explainable ai systems for the medical domain? *arXiv preprint arXiv:1712.09923* (2017).

[59] Doran, D., Schulz, S. & Besold, T. R. What does explainable AI really mean? A new conceptualization of perspectives. *arXiv preprint arXiv:1710.00794* (2017).

[60] Carvalho DV, Pereira EM, Cardoso JS (2019). Machine learning interpretability: A survey on methods and metrics. Electronics.

[61] Hinton GE, Osindero S, Teh Y-W (2006). A fast learning algorithm for deep belief nets. Neural computation.

[62] Lee, H., Pham, P., Largman, Y. & Ng, A. Y. Unsupervised feature learning for audio classification using convolutional deep belief networks. In *Advances in neural information processing systems*, 1096–1104 (2009).

[63] Auffray C, Chen Z, Hood L (2009). Systems medicine: the future of medical genomics and healthcare. Genome Med.

[64] Chen R, Snyder M (2013). Promise of personalized omics to precision medicine. Wiley Interdisciplinary Reviews: Systems Biology and Medicine.

[65] Emmert-Streib F, Dehmer (2018). A Machine Learning Perspective on Personalized Medicine: An Automatized, Comprehensive Knowledge Base with Ontology for Pattern Recognition. Mach. Learn. Knowl. Extr..

[66] Highnam G, Mittelman D (2012). Personal genomes and precision medicine. Genome Biology.

[67] Sarmiento, R. F. & Dernoncourt, F. Improving patient cohort identification using natural language processing. sssIn *Secondary analysis of electronic health records*, 405–417 (Springer, 2016). 31314253

[68] Mikolov, T., Chen, K., Corrado, G. & Dean, J. Efficient estimation of word representations in vector space. *arXiv preprint arXiv:1301.3781* (2013).

[69] Rumelhart DE, Hinton GE, Williams RJ (1988). Learning representations by back-propagating errors. Cognitive modeling.

[70] Mikolov, T., Deoras, A., Kombrink, S. & Burget, L. Empirical evaluation and combination of advanced language modeling techniques. In *Twelfth Annual Conference of the International Speech Communication Association* (2011).

[71] Pennington, J.Socher, R. & Manning, C. Glove: Global vectors for word representation. In *Proceedings of the 2014 conference on empirical methods in natural language processing (EMNLP)*, 1532–1543 (2014).

[72] Bengio Y, Ducharme R, Vincent P, Jauvin C (2003). A neural probabilistic language model. Journal of machine learning research.

[73] Yang, Z. *Deep Learning Methods for Patient Phenotyping from Electronic Health Records*. Master’s thesis, school Tampere University, Tampere University, https://trepo.tuni.fi/handle/123456789/27326 (2019).

[74] Zeiler, M. D. Adadelta: an adaptive learning rate method. *arXiv:1212.5701 arXiv preprint* (2012).

